# Is high sensitive-troponin I a reliable biomarker for cardiac injury in methadone toxicity? A prospective cross-sectional study

**DOI:** 10.1186/s40360-022-00558-6

**Published:** 2022-03-23

**Authors:** Hasan Shemirani, Masoumeh Sadeghi, Azadeh Davoudian Dehkordi, Farzad Gheshlaghi

**Affiliations:** 1 Department of Cardiology, School of Medicine, Isfahan University of Medical Sciences, Isfahan, Iran; 2 Isfahan Cardiovascular Research Center, Isfahan University of Medical Sciences, Isfahan, Iran; 3 Department of Clinical Toxicology, Isfahan Clinical Toxicology Research Center, School Of Medicine, Isfahan University of Medical Sciences, Khoorshid Hospital, Ostandari Ave., Isfahan, Iran

**Keywords:** Methadone, High-sensitivity troponin I, Electrocardiography, Poisoning, Echocardiography

## Abstract

**Background:**

Methadone is a synthetic opioid mostly used for detoxification therapy, as its use increases; the possibility for methadone-induced cardiotoxicity may rise. The aim of this study was to determine the association of high-sensitivity troponin I levels as a predictor of cardiac injury in methadone toxicity.

**Methods:**

Sixty methadone toxicity patients included in this prospective cross-sectional study from October 2018–November 2020. High-sensitivity troponin I level and electrocardiogram were assessed in patients at admission. All patients underwent echocardiography at admission and 30 days later and compared this finding between two groups based on high-sensitivity troponin I results.

**Results:**

Mean age of the patients was 34.5 ± 11.1 years (males: 67%). Twenty (20%) patients had positive high sensitive-troponin results. Long QT interval and inverted T in precordial leads were mostly observed in individuals with positive high-sensitivity troponin I (75% vs. 35%, *P* = 0.013 and 83% vs. 16%, *P* < 0.001, respectively). Patients with elevated troponin had reduced left ventricular ejection fraction in comparison to normal group during admission (43.1 ± 15.4% vs. 55%, *P* < 0.001) and this left ventricular ejection fraction remained abnormal after 30 days (43.7 ± 21.6%). Patients in positive high-sensitivity troponin I group had higher regional wall motion abnormality frequency both at admission and 30 days later compared to the other group (0 day: 42% vs. 0, *P* < 0.001, 30th days: 25% vs. 4%, *P* = 0.020).

**Conclusion:**

Patients with simultaneous methadone toxicity and positive high-sensitivity troponin I had worse cardiac outcomes and this biomarker could be probably used for better implementation of therapeutic interventions and prognosis.

## Introduction

Methadone is a synthetic opioid agent with analgesic properties used for severe pain management. Moreover, methadone is widely used in maintenance treatment of opioid addiction [1]. It has been reported that approximately 750,000 individuals are under methadone maintenance therapy (MMT) programs worldwide [2]. Due to the availability of methadone for MMT program, intentional and accidental poisoning is to be expected [1]. In recent years, Iran has perceived a shocking increase in the incidence of methadone poisoning, possibly due to easier access to opioids in adults and children [3–7].

According to DPIC’s reports methadone is one of the most common causes for admission of poisoned patients in the toxicology emergency departments in Iran [8].

Methadone is highly lipid soluble with 60 to 90% protein bounding. Its bioavailability ranges 70–90% and essentially eliminated via liver metabolism [9, 10].

Methadone is not a safe drug and in overdoses it causes severe central nervous system depression as well as skin, gastrointestinal tract, and urogenital and cardiovascular involvement. Cardiotoxicity is one of the most serious and lethal side effects of methadone poisoning. Complications such as increased QT dispersion, QT interval prolongation, and torsade de pointes (TDP), Takotsubo cardiomyopathy, and Brugada-like syndrome were reported [11, 12].

Methadone causes hemodynamic instability by decrease peripheral vascular resistance, heart rate, and decrease O2 saturation [13].

The cardiotoxicity of methadone is reported in the literature. Ehret et al. reported methadone increase QT interval in consumers rather than non-consumers (16.2% vs. 0, *P* < 0.001) and had correlation with daily dosage of methadone usage (r: 0.20, *P* < 0.01) [14].

Rahimdarabad et al. reported that coronary artery diseases were more prevalent in methadone user [15]. In another study authors indicated that opium usage could be as an independent risk factor for manifestation of cardiovascular diseases [16]. In contrast, Dehghani et al. concluded that the anterior myocardial infarction and related early mortality were lower in opium consumers [17].

hs-TnI is a reliable biomarker for detection of acute coronary syndromes. An abnormal ECG in the presence of a positive marker is strongly indicative of myocardial infarction (MI). A variety of diseases such as atrial fibrillation, sepsis, heart failure, myocarditis, pulmonary edema, renal disease, myocardial contusion, direct myocardial damage, coronary spasm, burns and acute stroke can elevate hs-TnI levels in the absence of acute coronary syndrome. On the contrary, hs-TnI is not specific for the acute thrombotic occlusion of a coronary artery [18].

The aim of this study was to determine the association of hs-TnI levels as a predictor of cardiac injury in methadone toxicity.

## Materials and methods

### Study design and setting

In a single center prospective cross-sectional study, all patients with methadone overdose admitted to Clinical Toxicology department of teaching Hospital between October 2018 to November 2020 were enrolled.

### Participants

All hospitalized adult and adolescents patients more than 16 years old with methadone poisoning, regardless of their age or sex, diagnosis based on history confirmed by positive urine test were included. We excluded patients with underlying liver or renal diseases, co-toxicity of drugs or other opioids, any past medical history of cardiovascular disease and chronic alcoholism or any diseases like diabetes mellitus that affect high sensitive Troponin (hs-TnI) value. After implementation of inclusion and exclusion criteria, a total of 60 patients were recruited in this study.

#### Data gathering

Demographic characteristics, occupation (employed/unemployed), cigarette smoking, Water pipe and alcohol as well as chronic methadone use were gathered through self-made questionnaire. At admission, heart rates (beats/minute), systolic/ diastolic blood pressure (mmHg), respiratory rates (rates/minute), O_2_ saturation (%), Hemoglobin (g/l), white blood cells (WBC) (^*^10^6^/l), blood urea nitrogen (BUN), Creatinine (Cr) (mg/dl) measured. According to severity of methadone toxicities, patients were distinct into three categories including mild (respiratory rates: 6–8/min and O_2_ saturation: 80–95%), moderate (respiratory rates: 4–6/min and O_2_ saturation: 60–80%) and severe (apnea and O_2_ saturation < 60%) [19]. hs-TnI (VIDAS, France) measured at admission and patients categorized to positive or negative groups. hs-TnI level of less than 0.04 considered negative, 0.04 to 0.06 borderline and more than 0.06 positive. ECG was recorded and the following parameters assessed: sinus tachycardia, inverted T in precordial and limb leads, long QT interval (males > 440 msec and females > 460 msec) and QRS variations. Echocardiography was performed for all patients (Samsung, hs40, 2017), cardiac condition and function included LVEF (%), diastolic dysfunction (%), regional wall motion abnormality (RWMA) (%), left ventricle size dilation (%), mitral regurgitation (MR) (%), tricuspid regurgitation (TR) (%) and pulmonary arterial pressure (PAP) (mmHg) were evaluated. Echocardiography was performed simultaneously with blood sampling and ECG. The same cardiologist reassessed participants 30 days later and measured all abovementioned cardiac parameters.

### Statistical analysis

The data were analyzed using Statistical Package for Social Sciences (SPSS) version 21 (IBM Corp., Armonk, NY, USA). Nominal and numerical variables were reported as frequency (percentage) and mean ± standard deviation, respectively. In order to evaluate the relation between categorical and continuous variables across different categories of hs-TnI at baseline and at the end of follow-up, we used chi-square and student-t test, respectively. Paired-t test and McNemar tests used for assessment of correlation between hs-TnI positive and negative groups in 1st and 30th day, as appropriate. A *P* value of less than 0.05 was considered statistically significant.

## Results

Sixty patients with the mean age of 34.5 ± 11.1 (range;17 to 58) years, 40 (67%) were men were included with methadone poisoning during the study period. All patients were in mild toxicity of methadone group. From the total of 60 recruited patients, 12 (20%) had positive High-sensitivity troponin I (hs-TnI) levels. General characteristics of study population based on positive or negative results of High-sensitivity troponin I (hs-TnI) are illustrated in Table 1. History of alcohol abuse was significantly higher (58% vs. 21%, *P* = 0.001), O_2_ saturation (88.6 ± 4.7% vs. 91.3 ± 2.6%, *P* = 0.012) was significantly lower and WBC means (11.9 ± 2.2 ^*^10^6^/l vs. 9.3 ± 1.8 ^*^10^6^/l, *P* < 0.001) were remarkably higher in positive group cardiac biomarker compared to the other group. Other laboratory data including Hb, BUN and Cr did not show any significant difference between two groups (Table 2).

**Table 1:** General characteristics of cases according to hs-troponin groups

Variables	Total (*n* = 60)	Hs-troponin positive (*n* = 12)	Hs-troponin negative (*n* = 48)	*P* Value
Age (years)	34.5 ± 11.1	37.1 ± 14.6	33.9 ± 10.2	0.369
Males N(%)	40 (67)	8 (67)	32 (67)	0.641
Unemployed N(%)	26 (43)	7 (58)	19 (39)	0.436
Cigarette smoking N(%)	38 (63)	8 (66)	30 (62)	0.789
Cigarette smoking duration (pack/year)	10.2 ± 15.9	15.6 ± 23.1	8.8 ± 13.5	0.186
Water pipe usage N(%)	31 (51)	7 (58)	24 (50)	0.605
Alcohol consumption N(%)	13 (21)	7 (58)	6 (12)	0.001
Methadone usage N(%)	30 (50)	8 (66)	22 (45)	0.197
Methadone usage duration^a^ (months)	3 (0, 33)	12 (0,54)	0 (0,24)	0.201
Methadone consumption^a^ (mg)	50 (21, 100)	50 (25,100)	50 (16, 144)	0.648

*Hs-Troponin* High sensitive troponin, *ECG* Electrocardiogram

^a^Both of the variables were not normally distributed so median (IQR) and Mann Whitney test have been used

**Table 2:** Vital signs and laboratory and electrocardiography findings according to hs-troponin groups on admission

Variables	Total (*n* = 60)	Hs-troponin positive (*n* = 12)	Hs-troponin negative (*n* = 48)	*P*
Heart rates (beats/minute)	100.0 ± 15.0	107.6 ± 15.7	98.1 ± 14.5	0.050
Systolic blood pressure (mmHg)	112.9 ± 10.5	110.9 ± 15.6	113.4 ± 9.0	0.472
Diastolic blood pressure (mmHg)	70.6 ± 8.2	70.2 ± 11.0	70.7 ± 7.5	0.846
Respiratory rate (rates/minute)	11.6 ± 1.4	11.0 ± 1.4	11.7 ± 1.3	0.123
O_2_ saturation (%)	90.8 ± 3.3	88.6 ± 4.7	91.3 ± 2.6	0.012
ECG finding (%)				
Normal	20 (33)	1 (8)	19 (39)	0.040
Sinus tachycardia	31 (52)	9 (75)	22 (46)	0.071
Inverted T in V1-V3	18 (30)	10 (83)	8 (16)	< 0.001
Long QT interval	26 (43)	9 (75)	17 (35)	0.013
Inverted T in II, III, avF	1 (1)	1 (8)	0	0.044
QRS variations	1 (1)	1 (8)	0	0.044
Hemoglobin (g/l)	143.5 ± 14.08	147.7 ± 18.33	142.5 ± 12.83	0.255
White blood cells (10^6^/l)	9.8 ± 2.1	11.9 ± 2.2	9.3 ± 1.8	< 0.001
Blood urea nitrogen (mg/dl)	26.7 ± 8.8	25.9 ± 9.2	26.8 ± 8.8	0.746
Creatinine (mg/dl)	0.84 ± 0.14	0.87 ± 0.17	0.83 ± 0.14	0.386

*Hs-Troponin* high sensitive troponin, *ECG* electrocardiogram

The ECG findings: The mean QT interval was 482.25 ± 19.57 msec in male and 490.05 ± 20.86 msec in female. Abnormal ECG findings including inverted T in V1-V3 (all three leads) leads and long QT interval were more prevalent in positive group (83% vs. 16%, *P* < 0.001 and 75% vs. 35%, *P* = 0.013, respectively). In contrast, normal sinus rhythm was mostly observed in those with negative hs-TnI. we don’t have any arrythmia except sinus tachycardia in this study.

Thirty patients (50%) used methadone for the first time in a suicide attempt. There was no statistically significant correlation between increased hs-TnI level with consumption of methadone for the first time or chronically use for Methadone Maintenance Treatment (MMT; data are not shown).

Table 3 figured various cardiac parameters in echocardiography based on hs-TnI results (positive – negative) on admission and 30th day. 42%of positive hs-TnI patients suffered from decreased LVEF)EF > 55.(%. Diastolic dysfunction was not significantly different between positive and negative hs-TnI case groups. Patients with positive hs-TnI level on admission significantly had lower LVEF and MR and higher left ventricular (LV) size dilation and tricuspid regurgitation (TR) (LVEF: 43.1 ± 15.4% vs. 55%, *P* < 0.001, MR: 50% vs. 63%, *P* = 0.041, LV size dilation: 42% vs. 0%, *P* < 0.001, TR: 50% vs. 15%, *P* = 0.008) in comparison with negative group. Alternatively, RWMA and PAP were surprisingly higher in positive group (42% vs. 0, *P* < 0.001 and 33.3 ± 9.3 mmHg vs. 26.9 ± 5.02 mmHg, *P* = 0.002). The RWMA was non-territory and mostly observed in anterior wall.

**Table 3:** Distribution of cardiac outcomes at baseline and 30 days after study initiation across different categories hs-troponin

Endpoint	Hs-troponin positive (***n*** = 12)		P*	Hs-troponin negative (***n*** = 48)		P*	***P***^***¶***^	***P***^***ƪ***^
	Baseline	After 30 days		Baseline	After 30 days			
**LVEF (%)**	**43.1 ± 15.4**	**43.7 ± 21.6**	**0.878**	**55**	**55.3 ± 1.2**	**0.083**	**< 0.001**	**< 0.001**
**Diastolic dysfunction (%)**	**3 (25)**	**2 (17)**	**0.317**	**4 (8)**	**4 (8)**	**0.346**	**0.108**	**0.389**
**Regional wall motion abnormalities (%)**	**5 (42)**	**3 (25)**	**0.483**	**0 (0)**	**2 (4)**	**0.511**	**< 0.001**	**0.020**
**LV size dilation (%)**	**5 (42)**	**2 (17)**	**0.375**	**0**	**0**	**1.00**	**< 0.001**	**0.004**
**Mitral regurgitation (%)**	**6 (50)**	**5 (42)**	**0.157**	**10 (63)**	**10 (21)**	**0.125**	**0.041**	**0.136**
**Tricuspid regurgitation (%)**	**6 (50)**	**4 (33)**	**0.572**	**7 (15)**	**7 (15)**	**0.553**	**0.008**	**0.133**
**Pulmonary arterial pressure (mmHg)**	**33.3 ± 9.3**	**32.9 ± 8.9**	**0.586**	**26.9 ± 5.02**	**28.9 ± 5.9**	**0.014**	**0.002**	**0.051**

*Hs-Troponin* high sensitive troponin, *LVEF* left ventricular ejection fraction, *LV* left ventricle

* *P* value between baseline and after 30 days

¶ *P* value between hs-troponin positive and negative group at baseline

ƪ *P* value between hs-troponin positive and negative group after 30 days

After 30 days, LVEF and RWMA were lower and higher in individuals with positive hs-TnI compared to negative ones (43.7 ± 21.6% vs. 55.3 ± 1.2%, *P* < 0.001 and 25% vs. 4%, *P* = 0.020) respectively. Although LVEF in patients with positive hs-TnI raised to approximately normal means from baseline but this correlation was not significant. We performed angiography on two patients in this study because of suspicious ECG changes indicating ischemia as well as their clinical status and positive hs-TnI.

*n*over all in first day of admission in echocardiography of 60 patieints: diastolic dysfunctio = 12%,RWMA = 8%,LV size dilation = 8%,MR = 27% and TR = 22%.In 30th day: diastolic dysfunction = 10%,RWMA = 8%,LV size dilation = 3%,MR = 25% and TR = 18%.

## Discussion

The aim of this study was to evaluate hs-TnI as a biomarker for cardiac injury in methadone toxicity.

In this study by electrocardiography and echocardiography it was shown that prevalence of long QT interval and inverted T waves in precordial leads in patients with positive hs-TnI was higher than negative group.

Echocardiographic findings showed that heart function was worse in the positive group than in the negative group.

Due to widespread usage of methadone in MMT programs and consequent raised prevalence of methadone toxicity, precautions should be made in terms of cardiovascular events. We did not found any positive findings in terms of other hemodynamic parameters including SBP, DBP or respiratory rates between groups this may be due to receiving treatment by EMS (emergency medical service (before being admitted to the hospital*.*

Endogenous ligands for opiate receptors were recognized in 1970s and these peptides were reported to affect cardiac tissue resulting in bradycardia, hypotension and peripheral vasodilation or even hypertension and tachycardia. Some records suggest that enkephalins play a primary role in some cardiac pathological circumstances including ischemic pre-conditioning with potassium-adenosine triphosphate channels located in myocardial cells mitochondria [20, 21]. The presence of sinus tachycardia in our study might be due to stimulation of these endogenous ligands.

Despite methadone is effective on all opioid receptors (κ, δ and μ), it basically act on μ receptor resulting in probable mimicking of endogenous opioids and block reuptake of substances including norepinephrine and serotonin in central nervous system [22, 23].

Methadone cardiotoxicity is reported in the literature. Ehret et al. found methadone prolonged QT interval among consumers rather than non-consumers (16.2% vs. 0, *P* < 0.001) and had correlation with daily methadone dosage (r: 0.20, *P* < 0.01) [24]. Methadone blocks human ether-a-go-go-related gene (hERG1) leading to prolongation of phase 3 of ventricular action potential resulting in long QT interval and possible occurrence of torsade de pointes [25]. Bradycardia induced by anticholinesterase characteristics of methadone or early after depolarization occurrence because of calcium and sodium-calcium exchange channels could be categorized as other possible mechanism for incidence of torsade de pointes [26, 27]. In our study prolonged QT interval was observed in 43% of cases and patients with positive biomarker had higher prevalence of this finding. It seems that myocardial injury induced by methadone might be responsible for higher frequency of long QT interval. Sheibani et al., found ECG abnormalities in all of dead cases with no correlation between ST-T abnormalities and coronary disease in autopsy [11]. In addition to rhythm disorders, methadone has been reported to be associated with other cardiovascular events. For instance, Najafi followed 566 patients with prior coronary artery bypass graft for 6.5 years and figured out patients with positive pre-operation opium usage had lower LVEF [28].

Although in our study LVEF of most of the patients became normal in 30th days of follow up but in some patients it remained lower, this finding might be due to close follow-up duration (thirty days). Additionally, the insignificancy of differences in LVEF between baseline and at the end of follow up among patients with positive high sensitive troponin might be due to small sample size. Considering that all patients with reduced LVEF had mild methadone toxicity, moderate and severe toxicity might be related with higher reduction in LVEF, so in severe cases of methadone toxicity this effect should be remember in mind in clinical settings.

Potential mechanisms of opium usage with occurrence of coronary artery diseases are reported in literature. Masoudkabir et al. reported that opium decreases plasma estrogen, testosterone, apolipoprotein A and increases insulin resistance, oxidative stress and inflammation, fibrinogen, factor VII as well as apolipoprotein B consequently leads to hypercoagulability status and increased likelihood of cardiovascular events [29]. However, we found no coronary lesions in patients underwent angiography. Gorgaslidze et al. enrolled 65 patients free of cardiovascular diseases who consumed opium and ephedrine. All participants underwent 24-h Holter monitor and ECG as well as echocardiography. They suggested that these individuals had reduced LVEF, ventricular dilation and systolic shortening of cardiac fibers revealed in echocardiographic findings and proposed the potential relation between opium and declined myocardial function [30].

In our study, 42% (*n* = 5) of patients with positive troponin had LV size dilation, this transient cardiomyopathy improved in most of the patients after 30 days. This phenomenon might be due to transient catecholamine surges induced by methadone usage ultimately caused takotsubo syndrome [30]. This stress cardiomyopathy mostly occurred due to emotional stress in presence of no obvious coronary artery lesions. A pediatric and adolescent cases have been reported to suffer from this type of cardiomyopathy [30, 31].

In our study, patients with positive hs-TnI had higher means of PAPs. The relation of methadone with raised pressure in pulmonary arteries has been suggested in experimental studies. Maiante et al. selected six dogs to evaluate the cardio-pulmonary effects of morphine and two different dosages of methadone. They found that PAPs were significantly higher in dogs received any dosages of methadone compared to morphine takers after 30 min of injection. This phenomenon might be related to decreased heart rate or vasoconstriction induced by methadone injection. Moreover, higher prevalence of smoking and possible occurrence of bronchiectasis might be other explanations of higher PAP means [32].

Till now there are few studies assessing the probable association of hs-TnI with methadone toxicity. Mostafavi et al. performed a cross-sectional study on 100 patients with simultaneous positive troponin and methadone toxicity to define the possible relation of this biomarker with probable coronary artery diseases. Their final findings suggested that raised hs-TnI levels in patients with methadone toxicity should not be defined as a manifestation of coronary artery syndromes [18]. Therefore, implementation of angiography must be performed based on patients’ clinical status as well as ECG alterations. We performed angiography on two patients in this study because of suspicious ECG changes indicating ischemia as well as their clinical status. Sheibani et al., studied on 245 pure methadone toxicity cases and found that hs-TnI had an independent significant association with mortality, with a cutoff value of 0.0365 ng/mL (odds ratio, 38.1; 95% CI, 2.3–641.9; *P* < 0.001) [33].

In current study, participants with positive troponin had higher WBC counts and lower LVEF compared to normal patients. This phenomenon might be partly explained by inflammatory cytokines and consequent effect on myocardial cells. Several complementary studies are required in this regard.

To best of our knowledge, current study is one of the the first in literature evaluating the relation of high sensitive troponin with ECG and echocardiographic findings in patients suffering from methadone toxicity.

It seems that methadone toxicity can lead to acute myocardial injury and should be investigated more in those with positive high sensitive troponin. However, by usage of other instruments including ECG, echocardiography and computed tomography (CT) coronary angiography as well as patient’s clinical status, implementation of invasive procedures could be prevented, especially during coronavirus pandemic.

In conclusion, this study indicates that ECG and cardiac function were worse among patients with concurrent methadone toxicity and positive high sensitive troponin.

Therefore, measurement of this biomarker could be effective for implementation of appropriate therapeutic interventions. Further studies are necessary confirming our findings.

### Limitations

Small sample size plus short follow-up duration might affect the generalization of our findings. According to our study, it was a novel study and there was no similar case for examining the number of samples. As a pilot study, 60 people were examined. Due to the prevalence of COVID19 in the world, the number of samples was limited. It is recommended that further studies be performed with a larger sample size (at least twice) The lack of information about diabetes among study participants was another limitation of our study [34]. Another limitation was that short follow-up duration might affect the interpretation of our results.

## Data Availability

The datasets generated during and/or analyzed during the current study are not publicly available due to confidential issues but are available from the corresponding author on reasonable request.

## Abbreviations

BUN: Blood urea nitrogen
CI: Confidence Interval
Cr: Creatinine
DBP: Diastolic blood pressure
DPIC: Drug and poison information center
ECG: Electrocardiogram
Hb: Hemoglobin
hs-TnI: High-sensitivity troponin I
MI: Myocardial infarction
MMT: Methadone maintenance therapy
MR: Mitral regurgitation
LV: Left ventricular
LVEF: Left ventricular ejection fraction
PAP: Pulmonary arterial pressure
QT: Q-T interval in electrocardiogram
RWMA: Regional wall motion abnormality
SBP: Systolic blood pressure
SPSS: Statistical Package for Social Software
TDP: Torsade de pointes
TR: Tricuspid regurgitation
WBC: White blood cell
